# Integrative network pharmacology, transcriptomics, and microbiomics elucidate the therapeutic mechanism of *Polygala tenuifolia* Willd water extract in chronic obstructive pulmonary disease

**DOI:** 10.3389/fmicb.2025.1703853

**Published:** 2025-11-25

**Authors:** Yiming An, Xiao Yu, Chao Wang, Xin Yu, Jingtong Zheng, Hongqiang Lin

**Affiliations:** 1 Department of Pathogen Biology, College of Basic Medical Sciences, Jilin University, Changchun, China; 2 Faculty of Basic Medicine, Department of Physiology, Daqing Medical College, Daqing, China; 3 Department of Histology and Embryology, College of Basic Medical Sciences, Jilin University, Changchun, China; 4 The Medical Basic Research Innovation Center of Airway Disease in North China, Ministry of Education of China, Changchun, China

**Keywords:** *Polygala tenuifolia* Willd, water extract, chronic obstructive pulmonary disease, transcriptomics, network pharmacology, gut microbiota

## Abstract

**Background:**

*Polygala tenuifolia* Willd (PT) is a plant with both medicinal and edible values. Traditionally, it has been used for sedation, enhancing cognition, resolving phlegm, and relieving cough. However, its protective effects and mechanisms against chronic obstructive pulmonary disease (COPD) remain unclear.

**Aim of the study:**

This study aims to observe the protective effects of the water extract of *Polygala tenuifolia* Willd (WEPT) on COPD, and to preliminarily elucidate its potential therapeutic mechanisms by integrating network pharmacology, molecular docking, multi-omics analysis, and molecular experiments.

**Methods and materials:**

HPLC quantified WEPT constituents. COPD mice models established via chronic smoke exposure underwent WEPT treatment, and the therapeutic effect was evaluated by lung function test, histopathology and cytokine profiling. Integrated multi-omics analyses (network pharmacology, transcriptomics, microbiomics) identified bioactive compounds, therapeutic targets, pathway regulations, and microbiota dynamics. Molecular docking validated compound-target interactions, while immunohistochemical/fluorescence assays confirmed key protein expression in lung tissues.

**Results:**

WEPT administration effectively reduced inflammatory cytokine levels in COPD mice, improved lung function, and alleviated histopathological damage like alveolar structural injury and airway inflammation. Network pharmacology and transcriptomic analyses identified Norhyoscyamine and Onjixanthone I as key active components, targeting PIK3CA and AKT1 via PI3K-AKT pathway regulation. Microbiome analysis showed WEPT restored gut microbiota balance. Molecular docking confirmed strong binding of bioactive compounds to core targets, while immunostaining assays demonstrated WEPT suppressed p-PI3K and p-AKT protein expression.

**Conclusion:**

WEPT may exert its intervention effects on COPD through a multi-target and multi-level comprehensive regulatory mechanism.

## 1 Introduction

Chronic obstructive pulmonary disease (COPD) is a progressive and commonly encountered respiratory illness characterized by persistent airflow limitation and chronic inflammation that builds up in the lung parenchyma and airways (Ferrera et al., 2021; Christenson et al., 2022; Agustí et al., 2023). COPD exhibits a high prevalence, morbidity, and mortality, ranking as the third leading cause of death globally (Obeidat et al., 2018; Halpin et al., 2019; Upadhyay et al., 2023). This disease imposes significant economic and social burdens and poses a substantial public health challenge (López-Campos et al., 2016; Chen et al., 2023). Although the pathogenesis of COPD remains incompletely understood, there is substantial evidence indicating that inhaling CS and/or other harmful particles increases oxidative stress and triggers inflammatory pathways (Austin et al., 2016; López-Campos et al., 2016; Brightling and Greening, 2019; Barnes, 2020). Chronic airway inflammation have long been recognized as playing pivotal roles in the development and progression of COPD (Gan et al., 2004; Barnes, 2014, 2016; Pavord, 2018). Of note, the inflammatory response triggered by CS and harmful particles extends beyond the lungs.

In recent years, increasing studies indicates that COPD is associated with the incidence of changes in intestinal health, in the meantime the dysbiosis of the gut microbiome plays a causal effect connected with the severity of CS-induced COPD pathogenesis (Budden et al., 2017; Bowerman et al., 2020; Lai et al., 2022; Karakasidis et al., 2023). Long-term exposure to CS has been reported to induce intestinal hypoxia and damage to intestinal cells, leading to gastrointestinal mucosal inflammation and alterations in the gut microbiota (Sze et al., 2014; Ahmad et al., 2017). Chronic exposure to CS can lead to a breakdown of the mucosal barrier and impaired intestinal permeability, allowing metabolites derived from gut microbiota, such as Short-Chain Fatty Acids (SCFAs), to enter systemic circulation. These SCFAs can then stimulate immune cells and cytokines, exacerbating lung inflammation (Meijer et al., 2010; Verdam et al., 2013; Trompette et al., 2014). These findings highlighted that inflammation in COPD was complicated and could involve multiple cascades. Aiming at a single target has limitations in clinical therapeutic efficacy. The current clinical medications used to treat COPD, including glucocorticoids and bronchodilators, are primarily intended to alleviate dyspnea resulting from bronchoconstriction. However, both have many adverse effects, and are difficult to halt disease progression or address inflammation in the small airways and lung parenchyma (Matera et al., 2011; Barnes, 2013; Brightling and Greening, 2019). Therefore, the anti-inflammatory drugs with multiple targets may represent a novel direction for managing the chronic inflammatory process associated with COPD.

Natural products harboring diverse bioactivities serve as pivotal reservoirs for the exploration of promising therapeutic compounds. Numerous studies have evidenced the capacity of Chinese herbal medicine to enhance proliferation and integrity of intestinal and lung epithelia, rebalance microbiota homeostasis, mitigate hyperimmune reactions, and confer advantageous impacts on the health of respiratory and gastrointestinal mucosae (Guo et al., 2017; Lai et al., 2022). *Polygala tenuifolia* Willd (PT) is a perennial herb in the family *Polygalaceae*. According to the *Chinese Pharmacopoeia* (2020 edition), PT is characterized by bitter-pungent flavor and warm property, with meridian entry specifically targeting the Heart, Kidney, and Lung systems (Xu et al., 2021). Its core pharmacological activities include intelligence-enhancing effects and phlegm-dissipating actions. Within traditional Chinese medicine (TCM) practice, it is systematically utilized in nootropic, expectorant, sedative, and anti-asthmatic formulations. For centuries, it has been utilized as an expectorant, ameliorating the symptoms of coughs, expectoration, bronchitis, asthma (El Sayah et al., 1999; Kamei et al., 2001; Lin et al., 2022; Zeng et al., 2022; Hao et al., 2024). Notably, modern fluid extract preparations of this botanical demonstrate significant therapeutic efficacy in alleviating bronchitis symptoms (Chen et al., 2024) and suppressing acute lung injury through anti-inflammatory mechanisms (Guo et al., 2024). Considering the above associations, PT may ameliorate COPD by suppressing the inflammatory response. Therefore, in this study, we evaluated the anti-COPD effect of WEPT and explored its possible mechanisms, aiming to provide experimental data supporting the expansion of medicinal applications and development of PT.

## 2 Materials and methods

### 2.1 Materials and reagents

The dried root of PT were purchased from pharmacies (origin: Jilin). It was identified as the root of PT by Professor Li Pingya, College of Pharmacy, Jilin University. Polygaxanthone III was purchased from Chengdu Pusi Biotechnology Co., LTD., and 3,6′-Disinapoyl sucrose was purchased from Vicchi Biotechnology Co., LTD.

### 2.2 Preparation of the WEPT

The PT root was dried, crushed, and sifted to obtain the powder of PT root, stored in the refrigerator at 4 °C. WEPT was prepared by taking 2 g of the PT root powder, adding 20 mL of purified water, soaking overnight at room temperature, ultrasonic extraction for two times (20 min each time), filtration, and then the filtrate was put into a rotary evaporator to obtain WEPT powder. The extract was stored in the refrigerator at 4 °C for preservation.

### 2.3 Analysis of chemical composition of samples

The powder of the WEPT was completely dissolved in purified water, and polygalaxanthone III and 3,6′-Disinapoyl sucrose were dissolved in methanol and filtered through a 0.45-μm filter membrane. Detection was carried out using an Acchrom S6000 HPLC-UV (Acchrom, China). Mobile phase: phase A was 0.05% aqueous phosphoric acid solution, and phase B was acetonitrile solution. The injection volume was set at 15 μL, the flow rate was 1 mL/min, the column temperature was set at 25 °C, and the detection wavelength was 320 nm. The elution gradient was as follows: 0 min–30 min, 10%–18%B; 30 min–60 min, 18%–26%B; 60 min–80 min, 26%–35%B.

### 2.4 Animal experimentation

#### 2.4.1 Establishment and treatment of animal models of COPD

Specific pathogen free (SPF) BALB/c mice, female, 8 weeks old, 18–20 g, were purchased from Animal Laboratory Center of Basic Medical College of Jilin University (Changchun, China). The animals were housed in the Animal Experimentation Center of the College of Basic Medical Sciences of Jilin University (SPF grade, Changchun, China). All experimental procedures involving mice management are strictly conducted in accordance with the guidelines outlined by the Experimental Animal Ethics Committee of Jilin University (Ethics Number 202407). All animals were kept in standard conditions with controlled humidity (50 ± 5%), temperature (22 ± 0.5 °C), and a 12-h light–dark cycle. After acclimatization and 2 weeks of rearing, they were tested. During the whole experimental period, the animals were given standard food, and allowed to drink water arbitrary. Besides their body weights were measured weekly. The study was conducted in a facility approved by the Institutional Animal Care and Use Committee of Jilin University, and the mice were maintained according to the Guide for the Care and Use of Laboratory Animals.

**Figure 1:**
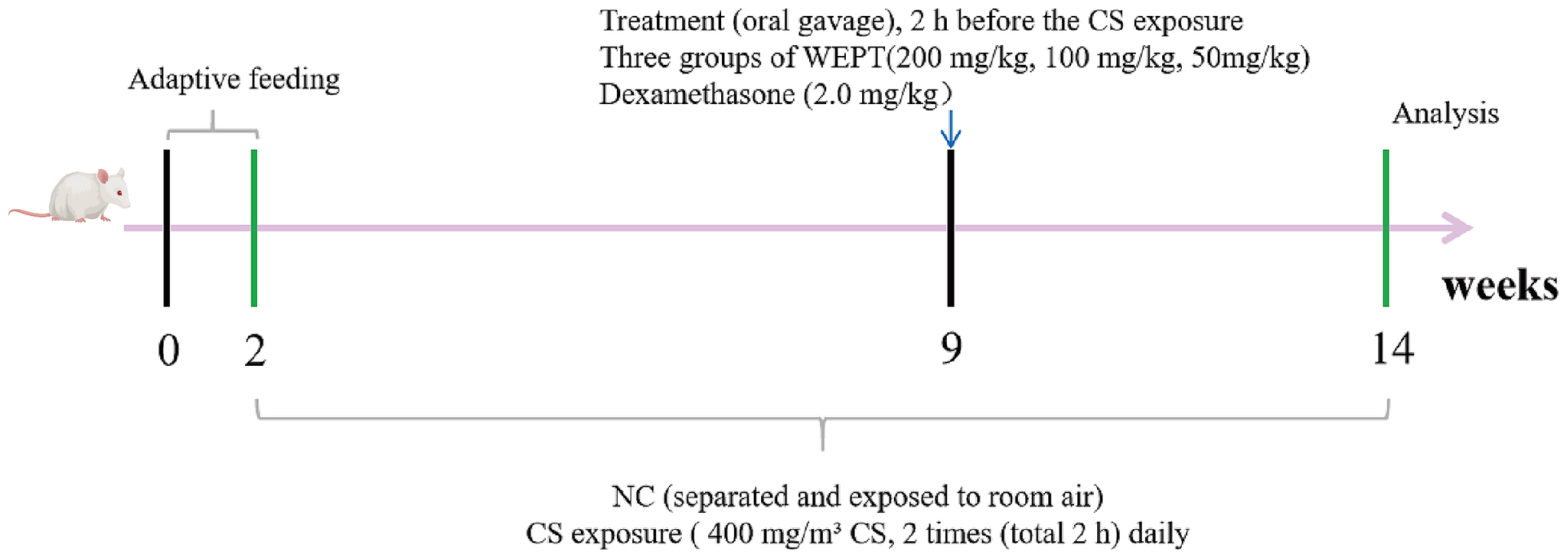
Experimental procedure flowchart.

After 2 weeks of adequate feeding, the mice were randomly divided into 6 groups (*n* = 15). These groups included the control group (NC group), the model group (CS group), CS exposure with WEPT high-dose treatment (HD group, 200 mg/kg/day), CS exposure with WEPT medium-dose treatment group (MD group, 100 mg/kg/day), CS exposure with WEPT low-dose group (LD group, 50 mg/kg/day), and CS exposure with dexamethasone treatment group (DEXA group, 2 mg/kg/day). The 15 mice in each group were randomly numbered 1 to 15. After fecal samples were collected for gut microbiota analysis, mice numbered 1–6 were assigned to transcriptomic and histopathological assessments (left lungs for RNA sequencing and right lungs for H&E/Masson staining). Mice numbered 7–12 were designated for invasive pulmonary function testing. The remaining mice 13–15 were allocated for ELISA, immunohistochemistry, and immunofluorescence assays. The mouse dosing regimen was determined using the body surface area (BSA) conversion method, following U.S. FDA guidelines for human-to-animal dose translation (Nair and Jacob, 2016). Based on the dose of raw *Polygala tenuifolia* Willd recommended by the Chinese Pharmacopoeia (v.2025), which ranges from 3 to 10 g, the calculated mouse equivalent dose range was 92.25–307.5 mg/kg. Accordingly, a medium dose of 100 mg/kg was selected, with a high dose of 200 mg/kg and a low dose of 50 mg/kg also established for this study. Passive smoking in mice is a well-established method for establishing COPD models (Campos et al., 2013; Lin et al., 2022). Therefore, the mouse passive smoking method was used in this study to establish a COPD model. Except for mice in the NC group, the mice of other groups were placed in a smoke exposure box (50 cm long, 40 cm wide and 30 cm high) connected to a peristaltic pump. The peristaltic pump was utilized to introduce CS into the exposure box. Each session involved the injection of smoke from 6 cigarettes, with a duration of 30 min per exposure. These exposures occurred twice daily, with an 8-h interval between each session, spanning 6 days a week, and persisting for a continuous period of 12 weeks. The mice in the NC group, which were not exposed to smoke stimulation, were placed in an independent box and exposed to the air. The successful establishment of the COPD model at week 9 was confirmed in a parallel validation cohort, with detailed results provided in Supplementary Figure S1. Between weeks 9 and 14, mice allocated to the HD, MD, LD, and DEXA groups received the designated dosage of the substance via oral gavage, administered 2 h prior to exposure to CS on a daily basis. The volume of drug administered was 10 mL/kg (Figure 1).

#### 2.4.2 Lung function tests

The lung function indexes of mice were detected using the spirometer (Spirometer: Buxco, PFT Controller, DSI, USA). The mice were anesthetized and endotracheally intubated, and the data were recorded after the mice had stabilized their respiration. The spirometer enabled the mice to passively inhale through the differential pressure flow sensor, converting the gas flow into electrical signals. These signals were then analyzed by a computer to obtain airway resistance (RI), forced expiratory volume in 100 ms (FEV100), forced vital capacity (FVC), forced expiratory volume in 1 s (FEV1), FEV100/FVC ratio, functional residual capacity (FRC), and other related parameters.

#### 2.4.3 Lung histopathology

Mouse lung tissue was perfused through the right ventricle with PBS to remove blood, following which the left lung tissue was excised and subsequently fixed in 4% paraformaldehyde. Following paraffin embedding and sectioning, the tissue samples were subjected to H&E staining. Microscopic examination revealed histopathological alterations including inflammatory cell infiltration, bronchial structural integrity, and bronchial epithelial thickness. Furthermore, Masson staining technique revealed collagen deposition in lung tissue, and the collagen deposition area was quantified using Image J software.

#### 2.4.4 Measurement of inflammatory factors in serum and lung tissue

Blood samples were collected from the retro-orbital plexus of mice, and serum was separated by centrifugation at 3,000 rpm for 10 min at 4 °C. Lung tissue samples were weighed, rinsed with ice-cold PBS, and homogenized. Following homogenization, the samples were centrifuged at 4 °C to collect the supernatant, in which the total protein concentration was quantified using a BCA assay for normalization. The concentrations of IL-1β, IL-6, and TNF-α in serum and lung homogenates were determined using commercial ELISA kits (Enzyme-linked Biotechnology Co., Ltd., China).

### 2.5 Network pharmacology

#### 2.5.1 Collection and screening of active chemical components and prediction of targets in PT

Main constituents of PT were identified via SymMap and supplemented with literature from CNKI and PubMed. Chemical structures and SMILES were retrieved from PubChem and SwissADME. Ingredients were screened for bioavailability, pharmacokinetics, drug likeness, and medicinal properties to select active ingredients. Active ingredient SMILES were input into Swiss Target Prediction to identify potential molecular targets.

#### 2.5.2 COPD target acquisition and construction of compound-disease-target network map

Using OMIM, MalaCards, and DrugBank databases with “COPD” as the keyword, we identified target genes. After merging and deduplicating the targets, we intersected PT and COPD targets to define core genes. A Venn diagram was created using an online tool. Data from PT chemistry, intersected targets, and COPD were compiled into data and type files, which were imported into Cytoscape 3.9.0 to construct a network illustrating the relationships among chemical components, COPD, and core targets. Topological analysis identified the top 5% degree-valued chemical components as key for COPD treatment in PT.

#### 2.5.3 PT-COPD intersection targets to construct PPI network maps

The intersecting targets of PT and COPD were imported into the STRING database to acquire the protein–protein interaction (PPI) network, setting the confidence interval to 0.70. Subsequently, the PPI network graph and related information were exported. The data were then imported into Cytoscape 3.9.0 for topology analysis of the network, acquiring relevant topological parameters. The core objectives were determined by filtering mean values greater than degree, proximity, and intermediate number.

#### 2.5.4 GO analysis and KEGG pathway enrichment analysis

The intersecting targets were submitted to the DAVID database^1^ for GO biological processes (BP), cellular component (CC), and molecular function (MF) analysis. Following literature review, the pathway most closely associated with COPD was identified from all enriched pathways as the focal pathway for this study. The selected pathways were then subjected to KEGG enrichment analysis using the bioinformatics mapping website, with a significance threshold set at *p* < 0.05 to generate the KEGG bubble map.

### 2.6 Transcriptomics analysis

RNA was extracted from the lung tissues of each group of mice using the TRIZOL method, and the quality of the extracted RNA was assessed. After the samples were qualified, mRNA was enriched using magnetic beads coated with Oligo (dt). Subsequently, the mRNA was fragmented by adding a fragmentation buffer. The fragmented mRNA was then used as a template to synthesize cDNA. Following this, the cDNA was purified using AMPure X beads to perform double-stranded cDNA purification and fragment size selection. Finally, the cDNA was enriched through PCR to obtain the final cDNA library. After quality control of the library, on-line sequencing was performed. After library quality control, online sequencing was conducted. The raw data obtained from sequencing were processed into sequenced reads using CASAVA Base Calling analysis. The read count data obtained from the gene expression level analysis were subjected to differential gene expression analysis, with the criteria for identifying differentially expressed genes set as *p* < 0.05 and |log_2_(foldchange)| > 1. Finally, the identified differentially expressed genes underwent GO, KEGG, and other enrichment analyses.

### 2.7 Comprehensive network pharmacology and transcriptomics analysis

To comprehensively and systematically investigate the potential mechanisms of WEPT on COPD, the results of network pharmacology and transcriptomics were integrated and analyzed. The intersecting targets identified by network pharmacology were analyzed in conjunction with the differentially expressed genes (DEGs) from transcriptomics using the DAVID database.

### 2.8 Molecular docking

The primary active ingredients of PT were identified through web-based pharmacological analysis for use as ligands. The structures of these key components were obtained from the PubChem database and converted into 3D structures using ChemBio3D Ultra 14.0 with energy minimization, serving as candidate ligands for molecular docking. Subsequently, the key targets identified through comprehensive analysis were employed as receptors. The binding affinity between the ligand and receptor was assessed based on the binding energy values obtained from the molecular docking results. Visualization of the results was performed using PyMOL software (version 2.3.5).

### 2.9 Immunohistochemical staining

Immunohistochemistry staining (IHC) was employed to assess the expression of p-AKT (Affinity Biosciences Cat# AF0016) and p-PI3K (Affinity Biosciences Cat# AF3241) in mouse lung tissues. Following microwave antigen retrieval for paraffin sections and inhibition of endogenous catalase activity with hydrogen peroxide, the sections were blocked with 3% BSA. Subsequently, overnight incubation with the primary antibody at 4 °C was carried out. The following day, sections were exposed to the secondary antibody at room temperature. Subsequent to diaminobenzidine (DAB) staining for color development and counterstaining with hematoxylin to visualize nuclei, the sections were examined and imaged using a 200 × microscope objective and analyzed using Image J software.

### 2.10 Immunofluorescence assay

For lung paraffin sections, antigen retrieval was performed following dewaxing in a 50 × sodium citrate buffer at 95 °C for 15 min, prior to immunofluorescence (IF) staining. Subsequent steps included serum blocking, incubation with a primary antibody (Affinity Biosciences Cat# AF0016), thorough washing, and nuclear staining with DAPI (Beyotime, China). Finally, the sections were stained and mounted with neutral resin. Fluorescence imaging was conducted using an upright microscope (BX-53, Olympus). Mean fluorescence intensity was quantified using Image J software.

### 2.11 Gut microbiota analysis

Fresh mouse feces from each group were collected, frozen in liquid nitrogen, and stored at −80 °C. Following the extraction of total fecal bacterial DNA from each group, specific primers with barcodes were synthesized based on the full-length primer sequence. Subsequently, the V3-V4 region of DNA was amplified via PCR, and the resulting products were purified, quantified, and homogenized to construct the sequencing library. The constructed libraries underwent thorough quality inspection, and only the qualified ones were subjected to sequencing using PacBio Sequel II. The effective data after screening are clustered at 97.0% similarity leThe screened effective data were clustered at a 97.0% similarity level to generate operational taxonomic units (OTUs). Based on the OTU results, flora identification, species distribution analysis, flora diversity assessment, and significant difference flora analysis were conducted for each group.

### 2.12 Statistical analysis

GraphPad Prism 9.5.0 software was applied for statistical analysis of data. Immunohistochemical grayscale values were analyzed using Image J software. One-way ANOVA was used for comparisons between multiple groups, and the LSD-t test was used for two-way comparisons between groups, and differences were considered statistically significant at *p* < 0.05.

## 3 Results

### 3.1 Quantification of the main ingredients of WEPT

**Figure 2:**
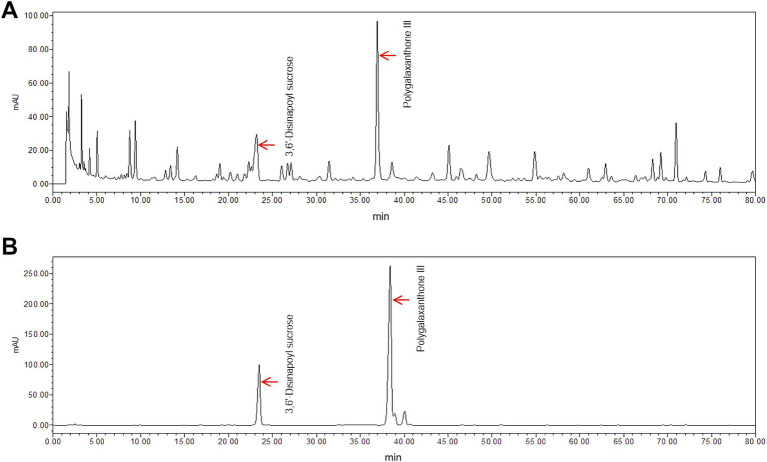
HPLC chromatogram (at 320 nm) of WEPT **(A)** and the reference substance **(B)**.

As shown in Figure 2, HPLC chromatogram of the main components of the WEPT and authentic standards. Based on the corresponding peak areas and concentrations of the two standards (standard curve *R^2^ >* 0.99), the main components of WEPT were quantified as follows: Polygalaxanthone III, 14499.225 μg/g, 3,6′-Disinapoyl sucrose, 37254.88599 μg/g. The content of Polygalaxanthone III in the WEPT was over 0.15%, and that of 3,6′-Disinapoyl sucrose was over 0.3%, which were in accordance with the requirements of Chinese Pharmacopoeia v.2020.

### 3.2 Anti-COPD activity

#### 3.2.1 Effect of WEPT on body weight of mice

**Figure 3:**
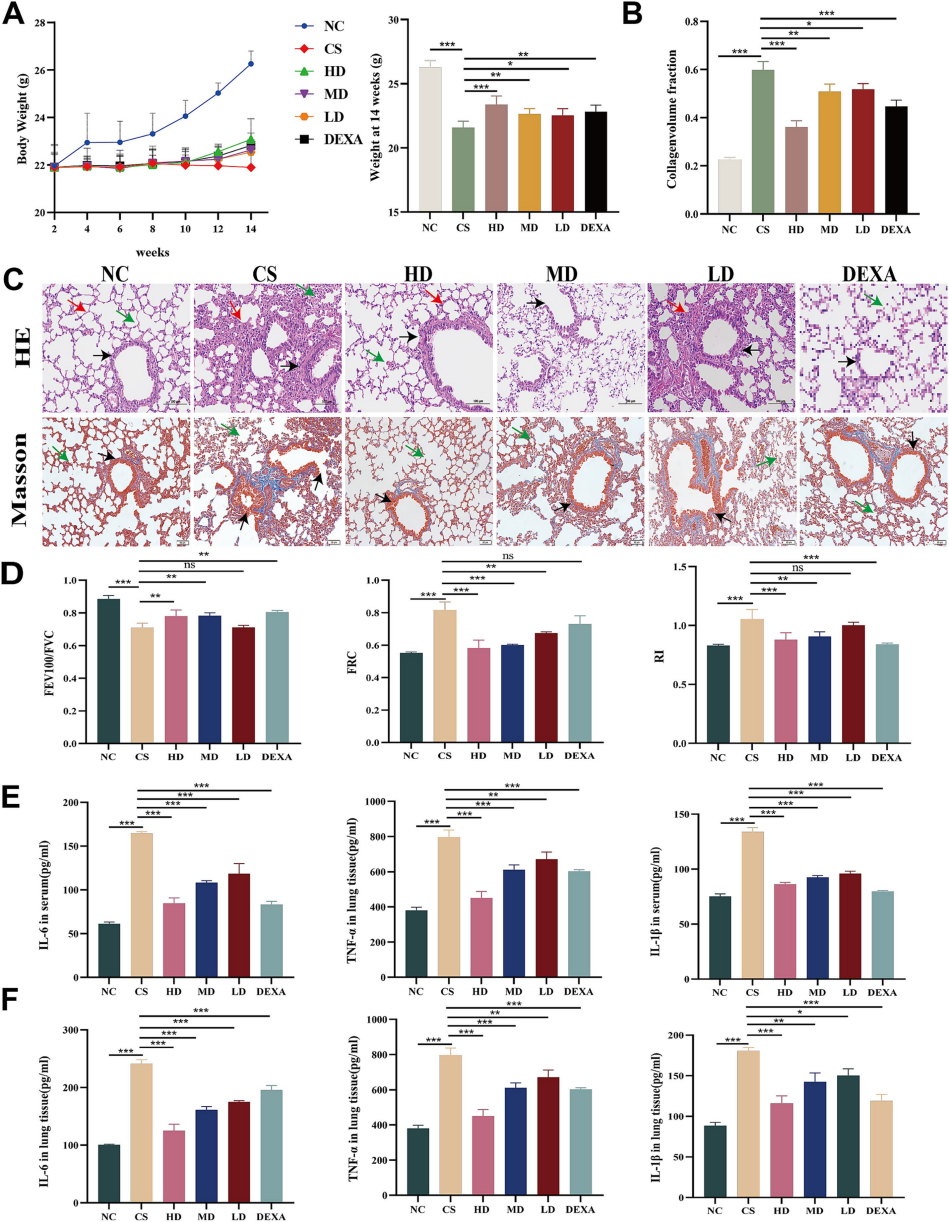
WEPT improves lung function, alleviates lung injury, and reduces inflammatory factor levels in COPD mice. **(A)** Weight change chart of mice from 2 to 14 weeks; **(B)** Collagen deposition area of mice in each group (*n* = 6); **(C)** H&E staining and Masson staining of mouse lung tissue (*n* = 6). In the context of pulmonary histopathology, the red arrows delineate areas of inflammatory factor infiltration. The green arrows highlight the thickening of the alveolar septum. Concurrently, the black arrows indicate the thickening of the bronchial wall; **(D)** FEV100/FVC (forced expiratory volume in 100 milliseconds/forced vital capacity), FRC (functional residual capacity), RI (airway resistance) (*n* = 6); **(E)** Inflammatory factor levels in mouse serum (*n* = 3); **(F)** Inflammatory factor levels in mouse lung tissue (*n* = 3); The results represented as mean ± SD. *p < 0.05, **p < 0.01, ***p < 0.001.

The body weight of mice was recorded weekly, as illustrated in Figure 3A. Mice in the NC group exhibited a gradual weight gain over the 1–14 week feeding period. Meanwhile, all mice in the experimental groups were subjected to 12 weeks of CS stimulation, with the exception of those in the NC group. From weeks 2 to 9, the weight of mice in all groups, except the NC group, declined, indicating a more effective establishment of the COPD model. Starting from week 9, WEPT and DEXA were administered via oral gavage. It was evident that the weight of mice in the experimental groups began to rise in comparison to those in the CS group (Figure 3A). The body weight of mice in the 14th week was analyzed, showing a significant increase in the HD, MD and DEXA groups compared to the CS group (*p* < 0.001, *p* < 0.01, *p* < 0.01). In summary, our study found that WEPT significantly improved the body weight of COPD mice.

#### 3.2.2 Effects of WEPT on lung function in mice

According to related studies, continuous exposure of mice to CS resulted in an increase in RI and FRC and a decrease in FEV100/FVC ratio in lung function (Xie et al., 2019; Lin et al., 2022). Lung function tests revealed that mice in the CS group exhibited a reduced FEV100/FVC ratio compared to the NC group (CS vs. NC, *p* < 0.001), along with elevated FRC and RI (both *p* < 0.001). Following HD intervention, the FRC and RI parameters were significantly restored (*p* < 0.001 vs. CS group), and the FEV100/FVC ratio showed marked improvement (*p* < 0.01 vs. CS group). In contrast, the LD group demonstrated only partial recovery of FRC (*p* < 0.01 vs. CS group), with no statistically significant regulatory effects observed on either FEV100/FVC ratio or RI (Figure 3D).

#### 3.2.3 Effects of WEPT on lung histomorphometry

Figure 3C illustrated lung histomorphometric changes. Within the NC group, no evident pathological alterations were detected. The airway mucosal epithelial structure appeared normal, with an intact bronchial epithelial cell structure, and the alveolar architecture remained relatively intact, showing no apparent inflammatory cell infiltration. In the CS group, exudates were present in the lumen of bronchial tubes and fine bronchioles. Alveolar rupture, fusion, and formation were noted, accompanied by conspicuous infiltration of inflammatory cells into the alveoli and local blood vessel congestion. Compared with the CS group, less inflammatory cell infiltration was observed in the DEXA group, and the alveolar structure remained intact. Within the HD group, there was a notable reduction in the degree of degeneration and necrosis of the tracheal epithelium, along with a significant decrease in inflammatory cell infiltration. The alveolar structure surrounding the fine bronchioles was distinctly delineated but remained partially dilated. While the morphological alterations in the alveoli were attenuated in the MD and LD groups, disruption of bronchiolar structure and inflammatory cell infiltration persisted. Masson staining is an effective method for assessing collagen fiber deposition in lung tissue. Compared to the control group, mice in the model group exhibited disrupted airway structures, significant thickening of the airway walls, substantial blue collagen fiber deposition around the airways, and narrowed airway lumens (Figure 3C). In contrast, the collagen fiber deposition area in the HD group was significantly reduced (*p* < 0.001) (Figure 3B), indicating that WEPT effectively decreased collagen fiber deposition in the airway.

#### 3.2.4 Effect of WEPT on IL-1β, TNF-α, and IL-6 levels

Cigarette smoke (CS) exposure induced systemic inflammatory responses, with significantly elevated serum levels of pro-inflammatory cytokines IL-6, TNF-α, and IL-1β in the CS group compared to the NC group (*p* < 0.001 for all). Compared to the CS group, both the HD and DEXA groups demonstrated significant suppression of these inflammatory mediators (*p* < 0.001 for IL-6 and IL-1β; *p* < 0.05 for TNF-α). The MD and LD groups also exhibited inhibitory effects on IL-1β and IL-6 expression (*p* < 0.001 vs. CS group), but showed no significant impact on TNF-α levels (Figure 3E).

Furthermore, analysis of lung tissue revealed that treatment with WEPT significantly and dose-dependently reduced the expression of pro-inflammatory cytokines (IL-6, TNF-α, and IL-1β) compared to the CS group (Figure 3F). These findings indicate that WEPT elicit not only a systemic anti-inflammatory effect but also confer specific protection to lung tissue.

### 3.3 Results of network pharmacology analysis

**Figure 4:**
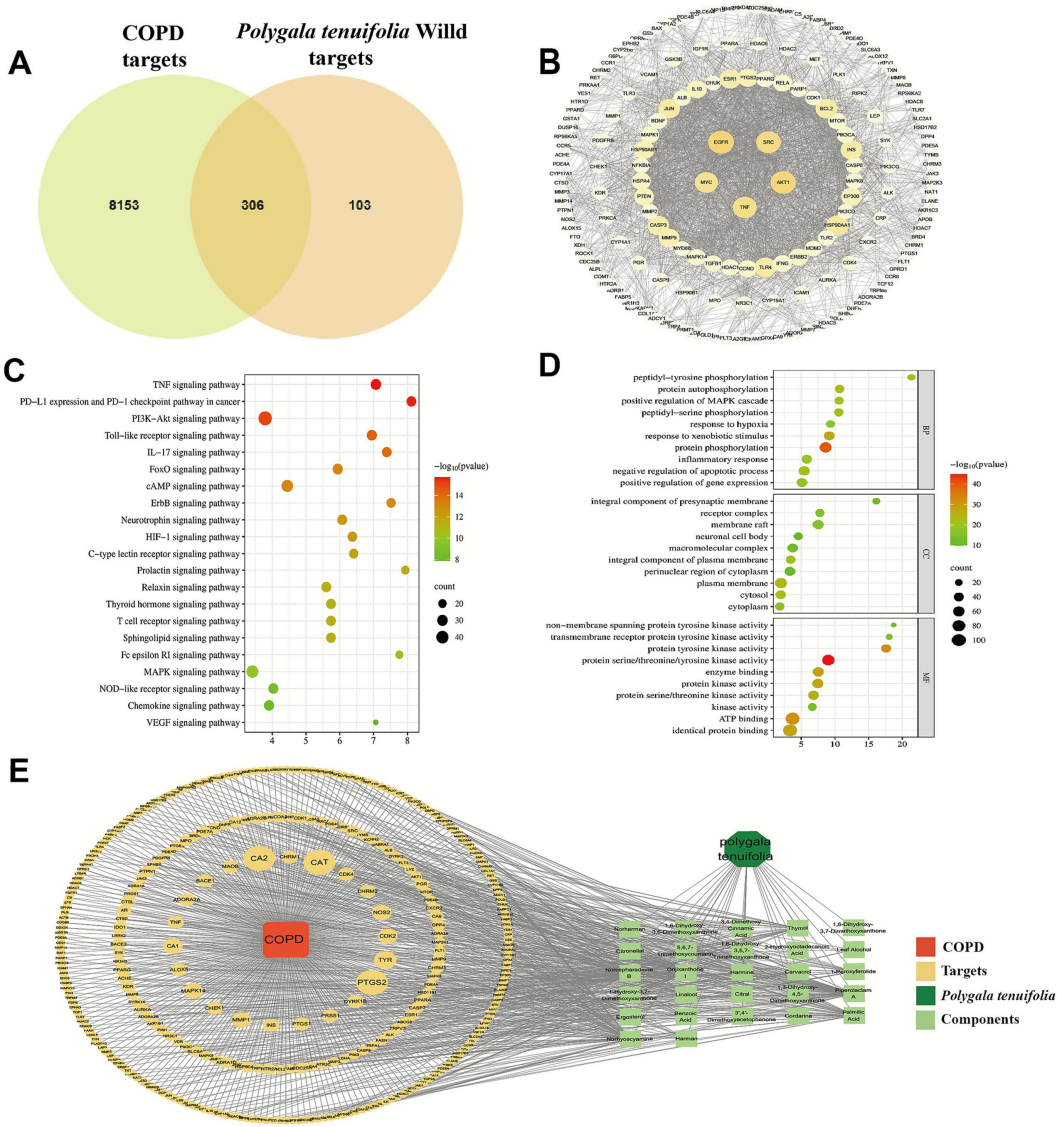
Network pharmacological results of *Polygala tenuifolia* in the treatment of COPD. **(A)** Intersection target of COPD and *Polygala tenuifolia*; **(B)** PPI network based on intersection targets; **(C)** KEGG enrichment based on intersection targets; **(D)** GO enrichment based on intersection targets; **(E)** “*Polygala tenuifolia*-components-targets-COPD” network.

The chemically active ingredients’ targets of PT, obtained post-screening, were overlapped with the targets associated with COPD, resulting in a total of 306 intersecting targets. The Venn diagram depicting this overlap was presented in Figure 4A. The 306 intersecting targets obtained were subjected to PPI network analysis using the STRING database. A minimum interactivity score of >0.700 was applied, resulting in 186 targets. Subsequently, the PPI network graph was generated using Cytoscape 3.9.0 to remove isolated nodes (Figure 4B). Network analysis identified genes with a degree value >20 as core targets (totaling 43), among which *EGFR*, *AKT1*, *SRC*, *MYC*, and *TNF* demonstrated potential close associations with the therapeutic mechanism of WEPT in COPD. KEGG analysis revealed significant enrichment of the TNF signaling pathway along with the PI3K-Akt signaling pathway among inflammation-related pathways (Figure 4C). GO enrichment analysis revealed that biological processes (BP) were predominantly linked to negative regulation of protein phosphorylation and apoptotic processes. Cellular components (CC) were primarily localized in the cytoplasm and plasma membrane. Molecular functions (MF) were mainly associated with protein binding, ATP binding, and protein serine/threonine/tyrosine kinase activity (Figure 4D). The obtained 27 PT active ingredients and the intersection targets of PT active ingredients and COPD targets were imported into Cytoscape 3.9.0.to construct the “*Polygala tenuifolia*-components-targets-COPD” network (Figure 4E). It is commonly accepted that components with a greater number of targets and higher degree values may be potentially significant. Therefore, key component screening was conducted by analyzing the degree value. The top five components (Degree >40) identified were: Onjixanthone I, Norhyoscyamine, Norcepharadione B, Piperolactam A and Palmitic Acid. Therefore, we speculate that these five components may play critical roles in the therapeutic effects of WEPT on COPD.

### 3.4 Transcriptomics analysis results

**Figure 5:**
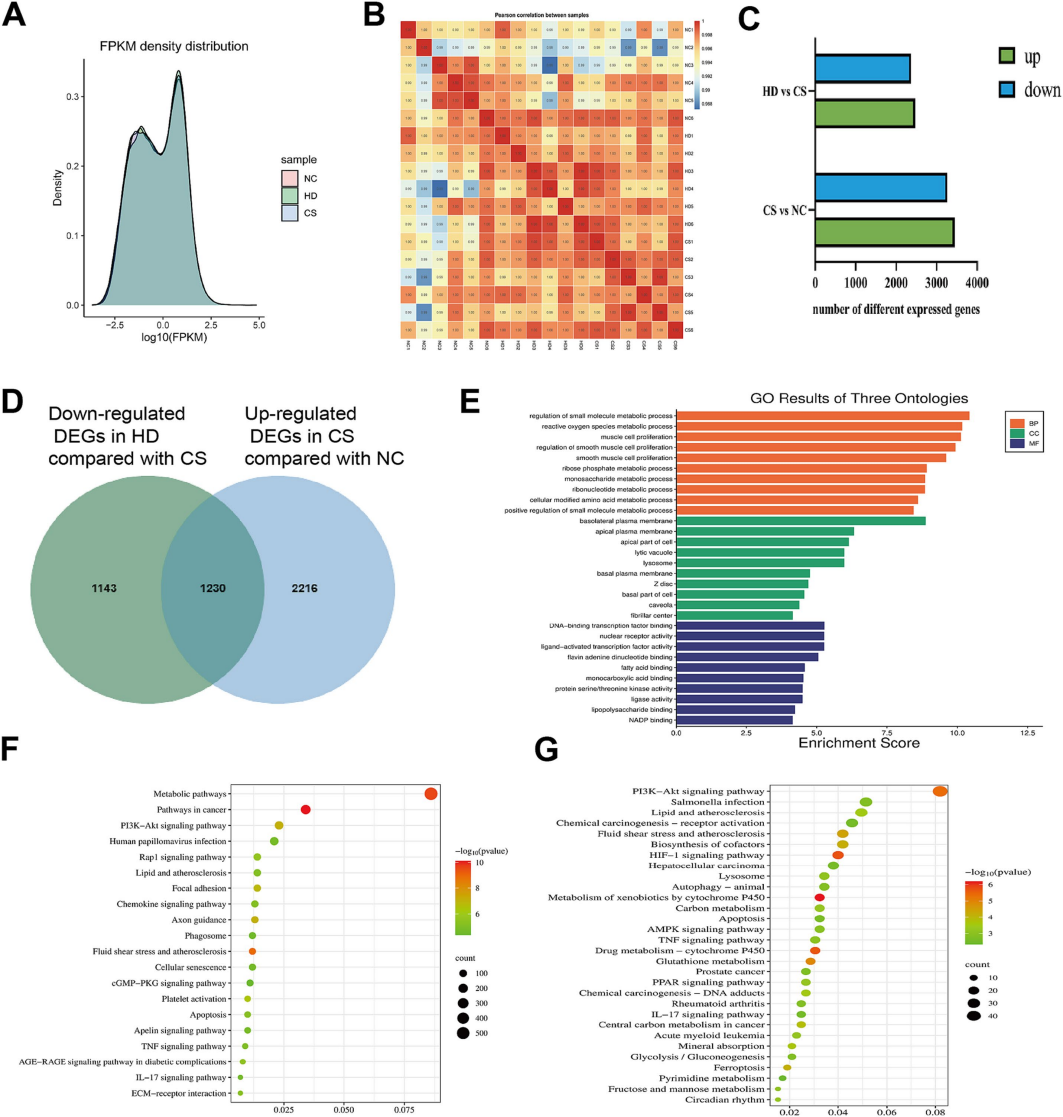
Transcriptomics analysis results of WEPT treated mice, CS group mice and NC group mice (*n* = 6). **(A)** FPKM expression diagram between different sample groups; **(B)** Heat map of correlation of gene expression levels between samples; **(C)** DEGs expression levels of HD vs. CS and CS vs. NC (*p* < 0.05); **(D)** Venn diagram of intersection genes up-regulated by CS stimulation and down-regulated by WEPT treatment; **(E)** GO enrichment analysis of intersection genes; **(F)** KEGG enrichment analysis of DEGs for NC vs. CS (Top 20); **(G)** KEGG enrichment analysis of intersection genes.

Transcriptomic analysis employing second-generation sequencing (NGS) technology was conducted to evaluate gene expression profiles. By analyzing Fragments Per Kilobase Million (FPKM) values, this approach not only enabled direct comparison of inter-sample gene expression differences but also effectively revealed the dispersion patterns of gene expression levels within individual samples, while providing a visual assessment of overall transcript abundance across sample groups. As demonstrated in Figure 5A, the normalized gene expression levels across the three experimental groups exhibited highly homogeneous distribution, robustly confirming the superior uniformity of the biological samples utilized in this study. This methodological rigor establishes a reliable foundation for subsequent precision analyses. Furthermore, regarding the correlation of gene expression levels between samples, Figure 5B illustrates that all correlation coefficients exceeded 0.99, suggesting that higher similarity between samples corresponds to increased accuracy and reproducibility of transcriptional results. In comparison to the NC group, the CS group exhibited 6,713 differential genes (DEGs), comprising 3,446 up-regulated genes and 3,267 down-regulated genes. Additionally, compared to the CS group, the HD group displayed 4,842 DEGs, with 2,469 up-regulated genes and 2,373 down-regulated genes (*p* < 0.05) (Figure 5C). Subsequently, KEGG analysis was performed on the DEGs of NC vs. CS, and the enrichment results showed (Figure 5F) that PI3K-Akt signaling pathway, TNF signaling pathway and IL-17 signaling pathway were significantly enriched. These pathways may be significant in the development of COPD in mice induced by CS. Our study centered on down-regulating genes, with the expectation that treatment with WEPT would alleviate the corresponding symptoms of COPD mice by down-regulating certain proteins or pathways. A Venn diagram was used to screen DEGs up-regulated in NC vs. CS and down-regulated in HD vs. CS (*p* < 0.05) (Figure 5D), and then GO and KEGG analysis were performed on these genes. KEGG enrichment analysis revealed significant enrichment of the PI3K-Akt signaling pathway, HIF-1 signaling pathway, AMPK signaling pathway, and TNF signaling pathway in the HD group (*p* < 0.05) (Figure 5G). At the same time, the analysis of enrichment results showed that WEPT may be involved in the regulation of small molecule metabolism, active oxygen metabolism and other important biological processes, and affected DNA-binding transcription factors, nuclear receptor activity and other molecular functions (Figure 5E).

### 3.5 Comprehensive network pharmacology and transcriptomics analysis results

**Figure 6:**
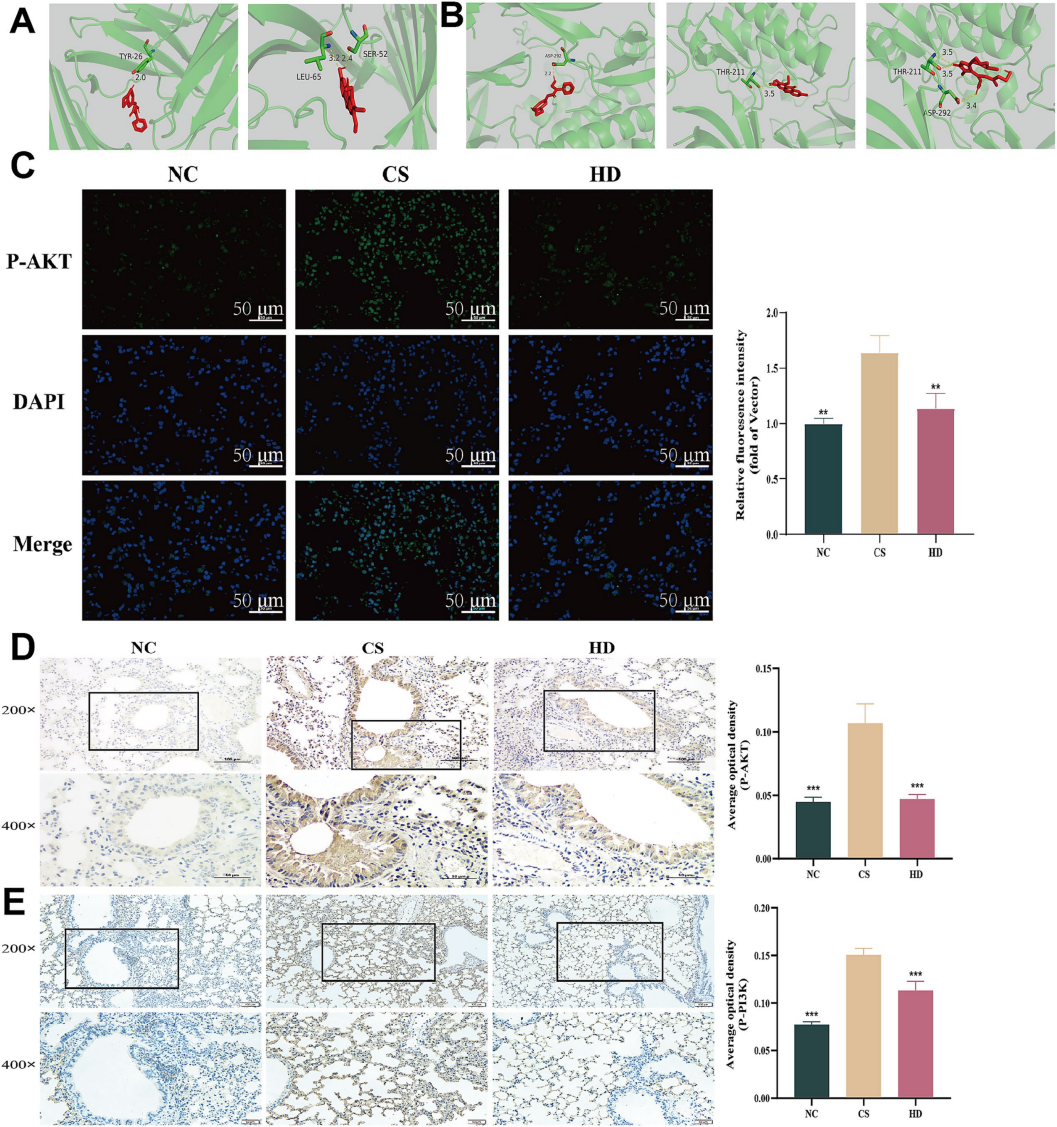
WEPT down-regulated the PI3K-Akt signaling pathway (*n* = 3). **(A)** The results of the docking of PIK3CA with Norhyoscyamine (binding energy: −9.1 kcal/mol), Onjixanthone I (binding energy: −9.1 kcal/mol); **(B)** Results of molecular docking of AKT1 with Norhyoscyamine (binding energy: −7.9 kcal/mol), Onjixanthone I (binding energy: −7.7 kcal/mol) and 1-Peroxyferolide (binding energy: −7.4 kcal/mol); **(C)** Immunofluorescence images of p-AKT and the corresponding relative fluorescence intensity (fold of Vector); **(D)** Immunohistochemical results of p-AKT; **(E)** Immunohistochemical results of p-PI3K. **p < 0.01, ***p < 0.001.

By integrating the potential pathways identified through network pharmacology with those uncovered in the transcriptomic study, we pinpointed the key targets and signaling pathways involved. Based on this integrative approach, the PI3K-Akt signaling pathway was identified as playing a pivotal role in the therapeutic effect of WEPT against COPD. Furthermore, by consolidating the network pharmacology results, we identified several specific compounds targeting the PI3K-Akt pathway, namely: Linalool, Onjixanthone I, Norcepharadione B, Thymol, 1-Peroxyferolide and Norhyoscyamine. Among these, Onjixanthone I and Norhyoscyamine were ranked among the top two compounds derived from *Polygala tenuifolia* in terms of their potential therapeutic contribution. In the network analysis, both PIK3CA and AKT1 exhibited degree values exceeding 20, indicating their high topological importance. It is worth noting that PIK3CA is a key catalytic subunit of the PI3K complex and serves as a canonical representative of PI3K regulatory and catalytic activity, suggesting its crucial role in the associated signaling cascade. To experimentally validate the interaction between the main bioactive compounds of WEPT and the core targets of the PI3K-Akt pathway, we performed molecular docking studies with AKT1 and PIK3CA. The key compounds selected for docking included Onjixanthone I and Norhyoscyamine (top-ranked compounds from network pharmacology), as well as 1-Peroxyferolide, which was specifically linked to AKT1 in the integrative analysis. The binding affinity between these compounds and the target proteins was quantitatively evaluated using binding scores, as illustrated in Figures 6A,B.

### 3.6 Effect of WEPT on PI3K-Akt signaling pathway

Immunohistochemistry staining was employed to validate the down-regulation of the PI3K-Akt signaling pathway, as indicated by the transcriptomics results. The results indicated an increase in the expression of p-AKT and p-PI3K proteins in the CS group. Following WEPT treatment, the protein expression levels of p-AKT (Figure 6D) and p-PI3K in the treatment group were lower compared to those in the CS group (Figure 6E). AKT, a pivotal regulator of the PI3K-AKT pathway, modulates diverse downstream signaling cascades by phosphorylating target proteins, thereby inhibiting or enhancing their activity. Additionally, AKT serves as a critical upstream modulator of oxidative stress and inflammation. Therefore, AKT was selected for further experimental validation. IF results confirmed a significant increase in fluorescence intensity within lung tissues of mice in CS group, indicative of substantial upregulation of p-AKT protein. This finding suggests that PI3K-Akt pathway is activated. Notably, this effect was markedly mitigated by treatment with WEPT (Figure 6C). This suggests that WEPT may ameliorate lung function and decrease the expression of inflammatory factors induced by CS through the down-regulation of the PI3K-Akt signaling pathway.

### 3.7 Gut microbiota analysis results

**Figure 7:**
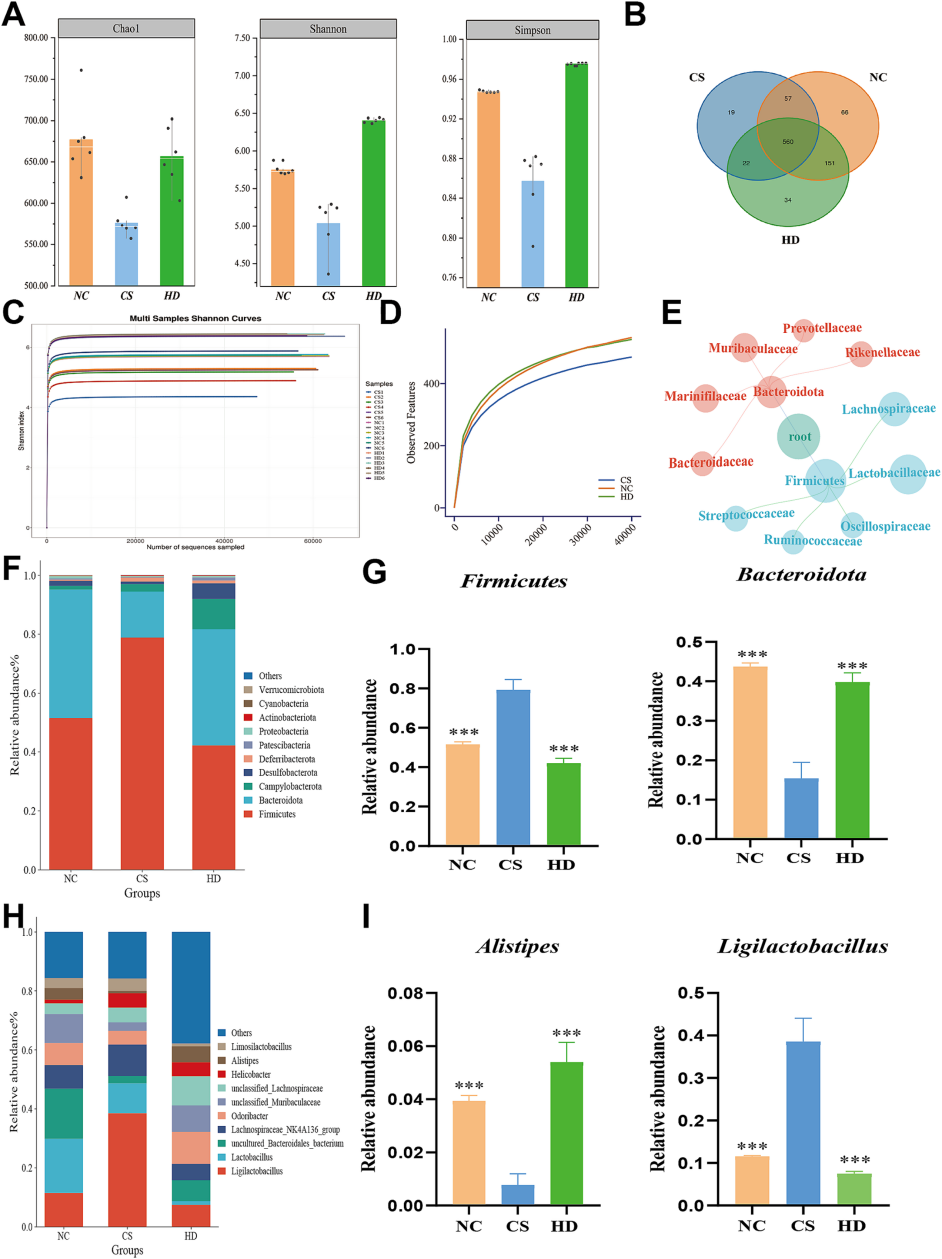
WEPT modulated the abnormal gut bacterial microbiota in COPD mice. **(A)** The results of *ɑ*-diversity (Chao1, Shannon, and Simpson indices) of the bacterial microbiota on the OTU level (*n* = 6); **(B)** Venn diagram of the similarity in composition on the OTU level among three groups (*n* = 6); **(C)** Shannon index (*n* = 6); **(D)** Rarefaction Curve (*n* = 6); **(E)** The families within *Firmicutes* and *Bacteroidetes* in the CS group (*n* = 6); **(F)** Bar chart of gut microbiota abundance in mice at phylum level (*n* = 6); **(G)** Relative abundance of *Firmicutes* and *Bacteroidetes* (*n* = 6); **(H)** Bar chart of gut microbiota abundance in mice at genus level; **(I)** Relative abundance of *Alistipes* and *Ligilactobacillus*. **p < 0.01, ***p < 0.001.

To evaluate the potential irritation caused by CS and the impact of WEPT treatment on alterations in gut microbiota, fecal samples from three groups of mice were collected and subjected to 16S rRNA sequencing analysis. In the analysis of *α*-diversity, by observing Chao1 Shannon and Simpson, it was observed that there was a lower trend in the CS group, compared to the NC and WEPT-treated groups (Figure 7A). The aforementioned three indices strongly suggest that CS-induced COPD in mice suppressed the abundance and diversity of gut microbiota, which were partially restored following WEPT treatment. The Venn diagram revealed 834, 658, and 767 common or unique OTUs in the NC, CS and WEPT groups (Figure 7B), respectively. Additionally, the three groups shared a total of 560 intersection OTUs. This result indicated that compare to the NC group, the number of differential OTUs in the CS group was low, while after the treatment of WEPT, the number of differential OUTs increased. A higher Shannon index indicates greater species diversity and abundance. The flattening of the curve in this study suggests that the majority of microbial species information in the sample has been captured (Figure 7C). Figure 7D indicates that the sequencing depth of each sample component was adequate. To further analyze the specific alterations in intestinal microbiota, the relative abundance of the predominant taxa (top 11) was examined using cluster analysis. At the phylum taxonomic level (Figure 7F), *Firmicutes* and *Bacteroidetes* were the most dominant phyla in all groups and exhibited significant changes. Compared to the NC group, mice in the CS group exhibited a notable increase in *Firmicutes* and a decline in *Bacteroidetes*, resulting in an elevation of the *Firmicutes/Bacteroidetes* ratio (Figures 7E,G). To observe changes in microbial composition, genus-level analysis was conducted. At the genus level (Figure 7H), independent of the other two groups, a significant decrease in the proportion of *Alistipes* was observed in the CS group. However, after WEPT treatment, these relative abundances of the bacterial microbiota were significantly reversed (Figure 7I). To be specific, the *Firmicutes/Bacteroidetes* ratio was slightly balanced which reveals that WEPT treatment could significantly improve microbial diversity and richness in COPD mice.

### 3.8 Multi-omics integrated analysis

**Figure 8:**
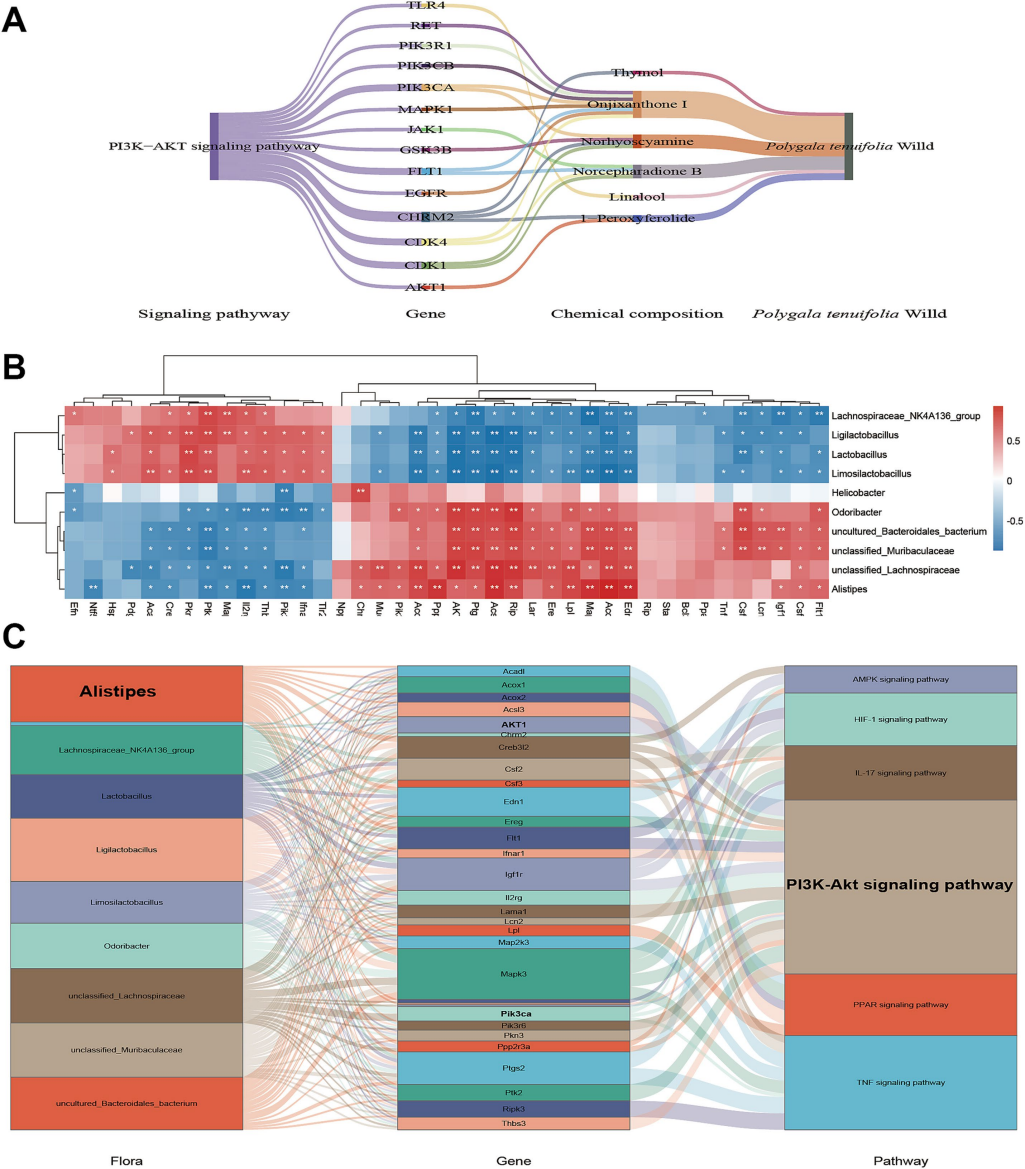
Integrative analysis reveals potential mechanisms underlying the therapeutic effects of WEPT in COPD. **(A)** The main compounds of WEPT act on genes in the PI3K-Akt signaling pathway; **(B)** Heatmap of correlation between DEGs and flora; **(C)** Flora-gene-pathway.

Network pharmacology and transcriptomics identified differential regulation of the PI3K-Akt signaling pathway, with components derived from PT emerging as key players. Notably, Onjixanthone I, a principal constituent of PT, showed significant enrichment, suggesting that it played a pivotal role in modulating the PI3K-Akt pathway (Figure 8A). Correlation analyses (*p* < 0.05) between differentially expressed genes and distinct bacterial communities were conducted, with the relationships visualized through heat maps (Figure 8B). A Sankey diagram was employed to delineate the “flora-gene-pathway” interactions (coefficients >0.65 or <−0.65). Our findings indicate that intestinal bacteria *Alistipes* and *Ligilactobacillus*, along with genes *Mapk3*, *Ptgs2*, and *Igf1r*, as well as the PI3K-Akt and TNF signaling pathways, are more closely associated with the therapeutic effects of WEPT in COPD (Figure 8C).

## 4 Discussion

COPD stands as a prevalent respiratory ailment characterized by small airway remodeling, mucus obstruction, and varying degrees of inflammatory response (Wang et al., 2018, 2020). Current clinical treatments for COPD primarily involve glucocorticosteroids and bronchodilators, however, they are often accompanied by side effects such as cardiac arrhythmia, increased susceptibility to infections, and metabolic disturbances (Tune, 2001; Burt et al., 2011; Liapikou et al., 2015). Thus, the quest for safer and more effective therapies is imperative.

This study specifically focused on *Polygala tenuifolia* (PT), a traditional Chinese medicine renowned for its effectiveness in cough suppression and anti-inflammatory properties, often incorporated into health foods. The pathogenesis of COPD remains unclear, and diagnosis relies mainly on symptoms. Selecting an appropriate animal model is crucial for smooth experiment progress. We chose a CS exposure method for female mice based on literature review (Lin et al., 2022; Upadhyay et al., 2023) and previous lab studies. As expected, after treatment with WEPT, the mice showed improvements in body weight, inflammatory factors, lung function indicators, and lung histopathology. These outcomes underscore the therapeutic potential of WEPT in alleviating the clinical manifestations of COPD. To elucidate the fundamental mechanisms of action at the molecular level, the high-dose WEPT group was selected for transcriptomic and gut microbiota analyses because it showed the strongest therapeutic effects. Focusing on the most responsive dose is cost-efficient for multi-omics studies and aligns with standard pharmacological practice (Song et al., 2024; Wang et al., 2024; Deng et al., 2025). Lower doses exhibited similar dose-dependent trends, indicating that high-dose analyses capture the core pharmacological profile of WEPT. Network pharmacology predicted key targets and pathways, such as PI3K-Akt, which were subsequently validated *in vivo* by transcriptomics. Concurrently, gut microbiota analysis indicated that WEPT remodels the microbial community (e.g., by promoting *Alistipes*), thereby contributing to efficacy via the gut-lung axis. These interdependent approaches collectively delineate a holistic picture of WEPT’s action, highlighting the pivotal role of the PI3K-Akt pathway and underscoring its therapeutic potential in restoring gut microbiota balance and combating COPD through the gut-lung axis.

Inflammatory responses play a pivotal role in the pathogenesis and progression of COPD. COPD is characterized by airway damage and abnormal pulmonary immune responses following exposure to CS and/or other pollutants, which are marked by the activation of neutrophils, macrophages, and an increase in the number of T lymphocytes (Th1 cells). Exposure to smoke leads to an increased release of the pro-inflammatory mediator IL-33 and alters the expression pattern of its receptor ST2, thereby silencing the function of innate lymphoid type 2 cells (ILC2) (Kearley et al., 2015). Furthermore, exposure to smoke can cause airway injury and alter the mucosal barrier function, making the airways more susceptible to infection. This promotes the conversion of ILC2 to ILC1, amplifying the inflammatory cascade (Barnes, 2016; Faiz et al., 2023; Riera-Martínez et al., 2023; Strickson et al., 2023). In this case, epithelial cells, neutrophils, and macrophages are activated to release a variety of inflammatory mediators, such as IL-6, IL-1β, and TNF-α. Current research indicates that IL-6 is a key biomarker for COPD, with its level changes being correlated with the severity of COPD and alterations in FEV1. TNF-α and IL-1β have been identified as pivotal cytokines with prominent roles in airway remodeling and the development of emphysema, capable of initiating inflammatory cascades during COPD exacerbations (Popa et al., 2007; Rincon and Irvin, 2012; El-Shimy et al., 2014; Farahi et al., 2017; Berry et al., 2022). In our study, treatment with WEPT resulted in a significant downregulation of elevated IL-6, IL-1β and TNF-α levels in the lung tissue, as well as IL-6, IL-1β and TNF-α in the serum of COPD mice, thereby providing evidence for its therapeutic potential in modulating inflammatory responses.

To explore the mechanism of WEPT in treating COPD, we combined predictions from network pharmacology with transcriptome sequencing of lung tissue from experimental mice. In our study, it was shown that the PI3K-Akt signaling pathway was activated in the CS group of mice, while WEPT treatment inhibited the expression of this pathway. The PI3K-Akt signaling is crucial in cellular regulation, affecting cell growth, proliferation, migration, metabolism, and secretion. The PI3K signaling transduction is one of the key pathways in virtually all cells. The PI3K signaling transduction is activated by a series of extracellular ligands, such as insulin-like growth factors (IGFs), which bind specifically to cell surface receptor tyrosine kinases (RTKs) or G protein-coupled receptors. Activated RTKs induce the phosphorylation of PI3K, which catalyzes the conversion of Phosphatidylinositol (4,5)-bisphosphate (PIP2) to phosphatidylinositol (3,4,5)-trisphosphate (PIP3). PIP3 serves as a second messenger, providing a docking site for Pyruvate dehydrogenase kinase 1 (PDK1) (Laplante and Sabatini, 2012; Yu and Cui, 2016; Jafari et al., 2019). Subsequently, PDK1 activates the protein kinase AKT/PKB through phosphorylation. The phosphorylation of AKT stimulates several downstream mediators of this signaling cascade and initiates various cytosolic events, such as cell survival, growth, proliferation, and differentiation. Some of these events are pro-inflammatory.

In the progression of COPD, the upregulation of PI3K and its downstream mediators, such as NF-κB and Matrix Metalloproteinase 9 (MMP-9), can excessively enhance the immune response and inhibit the function of proteins that exert antioxidant effects, such as Forkhead Box O (FOXO), and Sirtuin 1 (SIRT1), thereby inducing chronic inflammation (Yanagisawa et al., 2017; Moradi et al., 2021). AKT is a key protein kinase with a broad range of downstream targets in COPD. AKT activation is associated with PI3K activation. Once activated, AKT phosphorylates and activates its downstream proteins, regulating a variety of cellular signal transduction pathways. AKT inhibits the Histone Deacetylase (HDAC2), which can lead to increased inflammation and enhanced oxidative stress (Hosgood et al., 2009). In fact, most of the abnormally activated PI3K signaling pathways are associated with CS-induced airway inflammation and the pathogenesis of COPD. The activated PI3K signaling and its downstream mediators can cause further oxidative stress, thereby inducing progressive, persistent airway inflammation (Karrasch et al., 2008). In our study, we found that the key components of WEPT (Norhyoscyamine, Onjixanthone I, and 1-peroxyferolide) exhibit high binding affinity for AKT and PI3K proteins, suggesting that the therapeutic effects of WEPT are likely related to the PI3K-Akt signaling pathway. Furthermore, our immunohistochemical analyses revealed a suppression of p-AKT and p-PI3K protein expression within the pulmonary tissues of high-dose (HD) mice. This observation was corroborated by immunofluorescence assays, which demonstrated a significant increase in p-AKT protein levels in the CS group, contrasted by a notable reduction in the HD group. These findings suggest that a key mechanism by which WEPT treats CS-induced COPD is the blockade of the PI3K-Akt signaling pathway.

The mechanism underlying the anti-COPD effect of WEPT may not solely stem from the compounds absorbed by WEPT alone. Following oral consumption of WEPT, certain compounds inevitably remain in the intestine, which may directly or indirectly influence the abundance and diversity of intestinal microorganisms. The interplay between the lungs and gut microbiota creates a dynamic and interactive axis. The gut and lungs are interconnected and share a common endodermal embryonic origin, and there exists a “common mucosal response,” wherein the gastrointestinal mucosa may modulate pulmonary immune responses, and vice versa (Mestecky, 1987). The interplay between these two organs includes the transportation of microbial metabolites and the “spill over” of inflammatory mediators through the systemic circulation. Metabolites transported through the lung-gut axis, such as SCFAs, produced by gut microbiota and reaching the lungs through the systemic circulation (Ding et al., 2022), recruit and activate a large number of immune cells, such as promoting the differentiation of T cells into helper T cells (Th1 and Th17) effector cells, thereby enhancing pulmonary immune responses (Keely et al., 2012). Current studies indicate that patients with chronic respiratory diseases frequently exhibit gut microbiota dysbiosis. The intestinal microbiome may regulate pulmonary inflammatory responses and immune functions via the gut-lung axis, where dysbiosis may promote the production of pro-inflammatory mediators. These mediators can translocate into systemic circulation through a compromised mucosal barrier, exacerbating both pulmonary and systemic inflammation. This discovery resonates with the ancient Chinese medical doctrine *Huangdi Neijing (The Yellow Emperor’s Classic of Internal Medicine)*, which posits that “the lung and large intestine are exteriorly-interiorly paired.” This physiological-pathological correlation suggests a dynamic equilibrium system between the lung and intestine, wherein pathological cross-talk may occur during disease states. Consequently, modulating gut microbiota composition may represent a novel therapeutic strategy for chronic respiratory disorders.

An increasing number of studies recognize that the disruption of the gut microbiota is considered another key factor in the development of COPD. Previous studies on the gut microbiota in COPD have largely focused on the bacteria *Lachnospiraceae* and *Prevotella*, and research has confirmed that an increase in the abundance of *Lachnospiraceae* and *Prevotella* may be related to the exacerbation of COPD (Larsen, 2017; Li et al., 2021). Our 16S rRNA sequencing revealed that CS exposure induced profound gut microbiota dysbiosis, which was significantly ameliorated by WEPT treatment. A key alteration was the marked increase in the Firmicutes/Bacteroidetes (F/B) ratio in the COPD model group—a recognized hallmark of dysbiosis linked to inflammatory conditions. WEPT effectively normalized the F/B ratio, indicating restoration of microbial homeostasis. At the genus level, CS exposure led to a reduction in the beneficial bacterium *Alistipes*, while WEPT treatment notably increased its abundance. Given the reported role of *Alistipes* in maintaining intestinal mucosal integrity and mitigating inflammation, its restoration may help reinforce the gut barrier and reduce systemic dissemination of pro-inflammatory mediators (Tam et al., 2020; Liu et al., 2023; Wang et al., 2025). Conversely, we observed an expansion of the pathobiont *Helicobacter* in CS-exposed mice, which was suppressed by WEPT. This reduction in pro-inflammatory microbes provides a plausible link between gut microbiota modulation and attenuation of COPD-related inflammation (Wang et al., 2022). Notably, the enrichment of *Alistipes* coincided with the suppression of the PI3K-Akt signaling pathway in WEPT-treated mice. While no direct microbial-pathway interaction was established, we propose an indirect connection via attenuated systemic inflammation. Recent evidence suggests that *Alistipes* species can deliver anti-inflammatory metabolites, such as sulfonolipids, which suppress pro-inflammatory cytokines including IL-1β (Older et al., 2025). Since PI3K-Akt is a key signaling hub activated by inflammatory stimuli, a reduction in these cytokines could contribute to pathway downregulation. Thus, we hypothesize that WEPT-induced microbial remodeling—particularly the enrichment of *Alistipes*—works in concert with its direct pharmacological effects to dampen systemic inflammation and PI3K-Akt activation, collectively ameliorating COPD pathology.

However, this study has several limitations. First, the sample size remains limited, and future work with larger cohorts is needed to confirm the consistency of *Alistipes* reduction in COPD and its role in WEPT’s therapeutic mechanism. Second, the specific contribution of the PI3K-Akt pathway requires validation using selective inhibitors. Furthermore, the lack of bronchoalveolar lavage (BAL) to profile airway inflammatory cells hinder a more comprehensive correlation of local immune responses with systemic and tissue-level cytokine changes. Addressing these gaps in future studies will be crucial to refining our understanding of WEPT’s anti-COPD effects.

## 5 Conclusion

**Figure 9:**
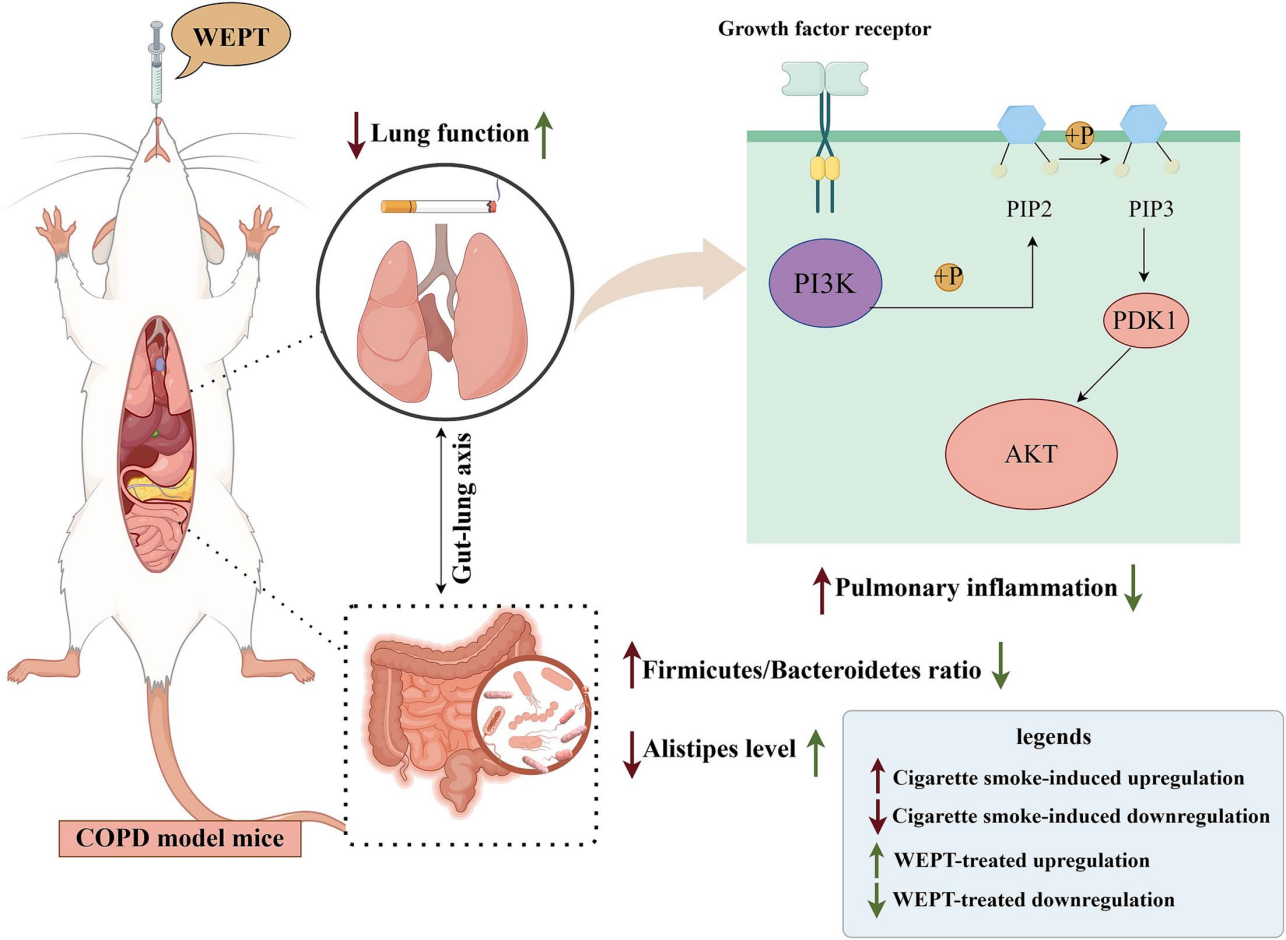
Schematic diagram of WEPT treatment in the COPD model: mainly through down-regulation of the PI3K-Akt signaling pathway to reduce inflammation, and remodeling of gut microbiota homeostasis (technical support by Figdraw).

In summary, we observed for the first time that treatment with WEPT significantly ameliorates symptoms in a CS-induced COPD mouse model. WEPT reduces pulmonary and systemic inflammation by inhibiting the PI3K-Akt signaling pathway. It also beneficially modulates the gut microbiome dysbiosis in COPD mice by enhancing the abundance of *Alistipes*. Our findings contribute to the broad medical application of *Polygala tenuifolia* Willd, introducing a novel phytotherapeutic approach for the treatment of COPD (Figure 9).

## Data Availability

The data presented in this study are publicly available. The data can be found at: https://ngdc.cncb.ac.cn, accession PRJCA046200.

## Footnotes

1
https://david.ncifcrf.gov/
