# Supplementary material for: Integrative network pharmacology, transcriptomics, and microbiomics elucidate the therapeutic mechanism of Polygala tenuifolia Willd water extract in chronic obstructive pulmonary disease
Source: Front Microbiol. 2025 Nov 25;16:1703853. doi: 10.3389/fmicb.2025.1703853 (PMC12685879; doi:10.3389/fmicb.2025.1703853)
Supplement: Supplementary file 1 [file Table_1.docx]

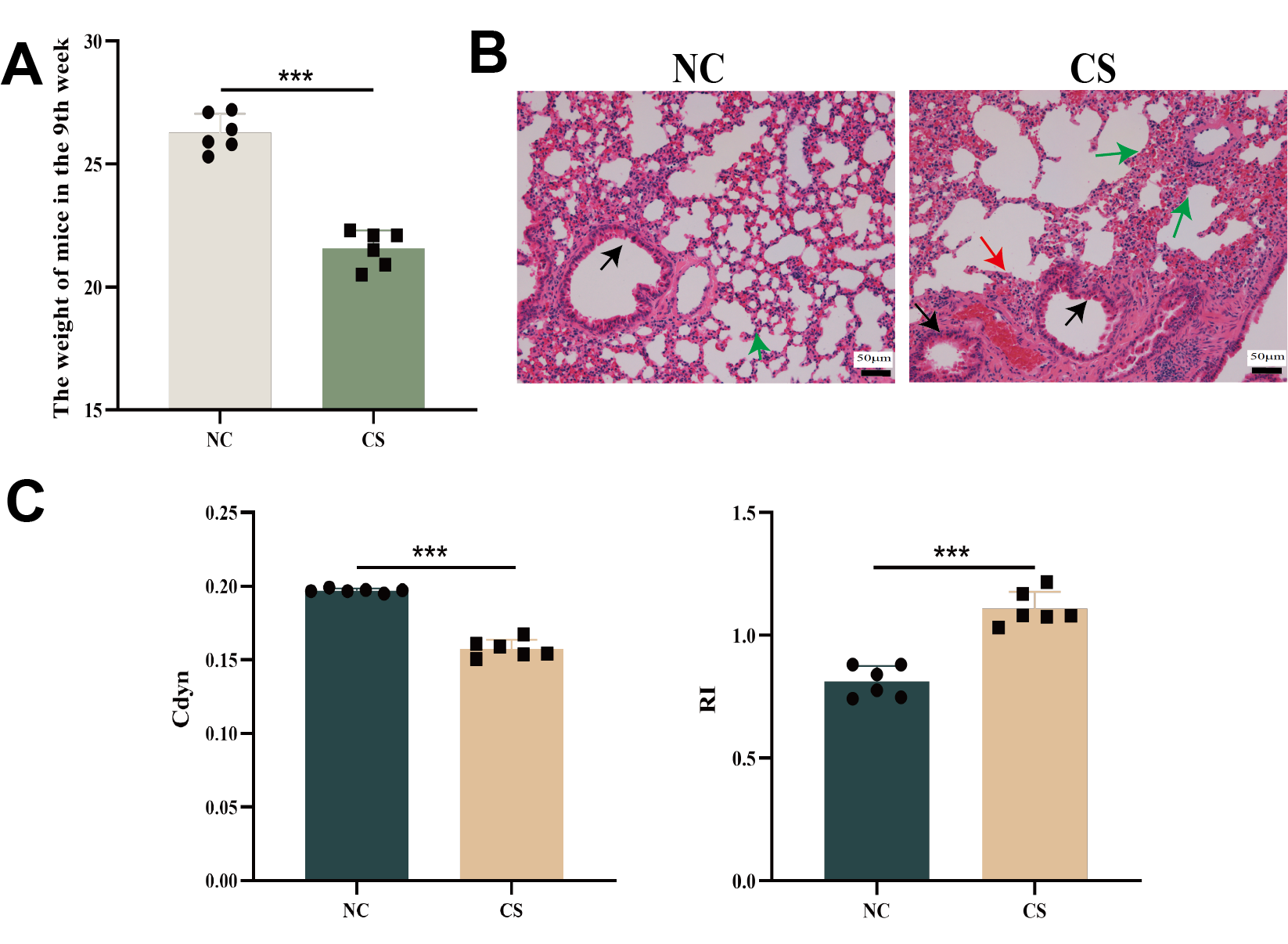


**Fig S1. Assessment of Key Parameters in the Validation Cohort (n=6) at Week 9.** A: Body weight of mice at Week 9; B: H&E staining of mouse lung tissue (n=6). The red arrows delineate areas of inflammatory factor infiltration. The green arrows highlight the thickening of the alveolar septum. Concurrently, the black arrows indicate the thickening of the bronchial wall; C: Cdyn (Dynamic lung compliance), RI (Airway resistance) (n=6). Data are presented as mean ± SD (n=6 mice per group). Statistical significance was determined by an unpaired two-tailed Student's t-test. ****P* < 0.001.
